# The Photoprotective Role of Spermidine in Tomato Seedlings under Salinity-Alkalinity Stress

**DOI:** 10.1371/journal.pone.0110855

**Published:** 2014-10-23

**Authors:** Lipan Hu, Lixia Xiang, Li Zhang, Xiaoting Zhou, Zhirong Zou, Xiaohui Hu

**Affiliations:** 1 College of Horticulture, Northwest Agricultural & Forest University, Yangling, China; 2 Key Laboratory of Protected Horticultural Engineering in Northwest, Ministry of Agriculture, Shaanxi Yangling, China; National Taiwan University, Taiwan

## Abstract

Polyamines are small, ubiquitous, nitrogenous compounds that scavenge reactive oxygen species and stabilize the structure and function of the photosynthetic apparatus in response to abiotic stresses. Molecular details underlying polyamine-mediated photoprotective mechanisms are not completely resolved. This study investigated the role of spermidine (Spd) in the structure and function of the photosynthetic apparatus. Tomato seedlings were subjected to salinity-alkalinity stress with and without foliar application of Spd, and photosynthetic and morphological parameters were analyzed. Leaf dry weight and net photosynthetic rate were reduced by salinity-alkalinity stress. Salinity-alkalinity stress reduced photochemical quenching parameters, including maximum photochemistry efficiency of photosystem II, quantum yield of linear electron flux, and coefficient of photochemical quenching (qP). Salinity-alkalinity stress elevated nonphotochemical quenching parameters, including the de-epoxidation state of the xanthophyll cycle and nonphotochemical quenching (NPQ). Microscopic analysis revealed that salinity-alkalinity stress disrupted the internal lamellar system of granal and stromal thylakoids. Exogenous Spd alleviated the stress-induced reduction of leaf dry weight, net photosynthetic rate, and qP parameters. The NPQ parameters increased by salinity-alkalinity stress were also alleviated by Spd. Seedlings treated with exogenous Spd had higher zeaxanthin (Z) contents than those without Spd under salinity-alkalinity stress. The chloroplast ultrastructure had a more ordered arrangement in seedlings treated with exogenous Spd than in those without Spd under salinity-alkalinity stress. These results indicate that exogenous Spd can alleviate the growth inhibition and thylakoid membrane photodamage caused by salinity-alkalinity stress. The Spd-induced accumulation of Z also may have an important role in stabilizing the photosynthetic apparatus.

## Introduction

Salinity-alkalinity stress is becoming one of the most serious abiotic stresses affecting crop growth and yield [1], and causes considerable economic losses. Our previous studies clearly show that plants are more sensitive to salinity-alkalinity stress than to neutral salt stress [1], [2]. Previous reports show that salinity-alkalinity stress strongly inhibits assimilation of photosynthetic carbon [3]–[5], which leads to an unfavorable dissipation of excess light energy. When photosystem II (PSII) is exposed to excess light, reactive oxygen species (ROS) such as singlet oxygen (^1^O_2_), hydroxyl ions (OH•), and H_2_O_2_ are formed by the interaction of molecular oxygen and triplet chlorophyll [6], [7], which causes photoinhibition and photodamage [8]. ROS directly affects chloroplasts and inhibits the repair of photodamaged PSII [7]. Salinity stress reduces photosynthesis and electron transport activity, thereby exacerbating problems arising from photoinhibition and photodamage of the photosynthetic apparatus [9].

Polyamines (PAs) are organic, low molecular weight, nitrogen-containing compounds; they include spermidine (Spd), spermine (Spm), and their diamine obligate precursor putrescine (Put) [10]. PAs function as plant growth regulators or intracellular messengers that regulate plant growth, development, and responses to abiotic stresses [11]. They also function as antioxidant, scavengers of ROS and free radicals [12], inhibit lipid peroxidation reactions [13] due to a combination of their anion- and cation-binding properties [14], and respond to a wide range of abiotic stresses. PAs also stabilize the structure and function of the photosynthetic apparatus in response to unfavorable environment factors [15]–[17]. PAs protect the structure of chloroplasts by keeping the thylakoid membranes in an orderly arrangement and maintaining high photosynthetic efficiency [18]. Exogenous Spd may regulate the synthesis of xanthophyll components and promote the conversion of violaxanthin (V) to zeaxanthin (Z), which can protect LHCII reaction centers against salinity-induced oxidative damage and injury from excess light energy [19].

The xanthophyll cycle pigment Z is formed from V via the intermediate anthoxanthin (A) during abiotic stress. Z is thought to be involved in photoprotective thermal dissipation and nonphotochemical quenching (NPQ) [20], [21]. The thermal dissipation process protects PSII against photodamage by decreasing excess redox potential of PSII and the electron transfer chain [22]. Z has an important antioxidant function in the lipid phase of the membrane and protects chloroplasts from photo-oxidative stress [23]. Z reduces the formation of ROS, or detoxifies already-formed ROS, to minimize photo-oxidative damage of the photosynthetic apparatus [21].

Previous studies showed that exogenous application of PAs partially alleviated the salinity-induced decline in photosynthetic efficiency [24]. However, the molecular mechanism of Spd-mediated photoprotection of the photosynthetic apparatus and the relationship between Spd and the xanthophyll cycle in response to salinity-alkalinity stress were not completely resolved. Therefore, we sought to determine whether exogenous Spd could protect tomato seedlings from photoinhibition and photodamage and interact with the endogenous xanthophyll cycle under salinity-alkalinity conditions.

## Materials and Methods

### Plant Materials and Growth Conditions

Tomato (*Solanum lycopersicum* cv. Jinpengchaoguan) seeds were germinated at 27°C in Petri plates lined with two layers of filter paper moistened with sterile distilled water. The germinated seeds were then sown in commix medium (Xintiandi Co., Yangling, Shanxi, China). Seedlings were grown in a controlled environmental greenhouse at Northwest A & F University, where the air temperature was maintained at 25±1°C/15±1°C (day/night). A 12-h light/12-h dark photoperiod was imposed with a daylight intensity of 800 µmol⋅m^−2^s^−1^. Seedlings were transferred into 40-L containers of one-half-strength Hoagland solution (pH 6.5±0.1; electrical conductivity, 1.4−1.8 ds⋅m^−1^; dissolved oxygen, 6.0±0.2 mg⋅L^−1^) when the third true leaves were fully expanded.

### Salinity-alkalinity Conditions and Spermidine Treatment

When seedlings had five true leaves, they were treated with 75 mM complex neutral and alkali salts [NaCl:Na_2_SO_4_:NaHCO_3_:Na_2_CO_3_ = 1∶9∶9∶1] and the foliage was sprayed with 0.25 mM Spd. The experiments included the following four treatments: 0 mM salinity-alkalinity plus 0 mM Spd (CK); 0 mM salinity-alkalinity plus 0.25 mM Spd (CS); 75 mM salinity-alkalinity plus 0 mM Spd (S); and 75 mM salinity-alkalinity plus 0.25 mM Spd (SS). Each treatment group included 48 plants and four replicates. Plant containers were arranged in a randomized block design. The solutions were renewed every 3 days. All experiments were performed at least three times with similar results.

### Plant Growth Measurements

Four days after the salinity-alkalinity treatment, plants were washed with sterile distilled water and dissected into leaves, stems, and roots. The fresh weights of dissected tissues were determined. The leaves, stems, and roots were dried at 105°C for 15 min and then at 75°C for 72 h to constant mass, and their dry masses were recorded. The shoot weight was equal to the sum of the leaf weight and the stem weight. The root/shoot ratio (R/S) was calculated as (R/S) = root weight/shoot weight. Four plants were measured per treatment.

### Gas-Exchange Measurements

Gas-exchange parameters were measured using a portable photosynthesis system (LI-6400, LI-COR Inc, USA). The photosynthetic rate was measured at 380±10 µmol⋅mol^−1^ CO_2_, 25°C, 70% relative humidity, and 800 µmol⋅m^−2^s^−1^ light intensity. Stomatal limitation (L_s_) was calculated as L_s_ = 1−C_i_/C_a_ (C_i_ and C_a_ represent the intercellular and ambient CO_2_ concentration, respectively) according to Farquhar et al. [25].

### Chlorophyll Fluorescence Parameters

Chlorophyll fluorescence was measured using a modulated fluorometer (PAM-2500; Walz, Effeltrich, Germany). The minimal (F_o_) and maximal (F_m_) Chl fluorescence emissions were determined after 30 min of dark adaptation, and F_o_′, F_s_, and F_m_′ were measured after light adaption at 600 µmol⋅m^−2^s^−1^. Fluorescence parameters were calculated as follows: maximum quantum yield of PSII, (F_v_/F_m_) = (F_m_−F_o_)/F_m_; maximum photochemistry efficiency of PSII, (F_v_′/F_m_′) = (F_m_′−F_o_′)/F_m_′; PSII operating efficiency, ΦPSII = (F_m_′−F_s_)/F_m_′; coefficient of photochemical quenching, Qp = (F_m_′−F_s_)/(F_m_′−F_o_′); and nonphotochemical quenching, NPQ = (F_m_−F_m_′)/F_m_′ [26].

### Pigments Analysis

Pigment composition was determined according to the procedure of Chen et al. with some minor modifications [27]. Xanthophyll pigments were extracted with 100% acetone under weak light. The extract was centrifuged at 12,000×*g* for 10 min. The supernatant was passed through a 0.2-µm syringe filter into an amber HPLC vial for immediate analysis. Twenty-five microliters of the filtered supernatant was analyzed with a Shimadzu LC-20A HPLC system (Kyoto, Japan). The Waters Spherisorb ODS1 column (5.0-µm particle size, 4.6×250 mm, Waters, USA) was used in the separation at 35°C. Solvent A [acetonitrile:methanol:50 mM Tris-HCl, 72∶8∶3 (v/v/v), pH 7.5] was used for the first 10 min, followed by a 2.5-min linear gradient to 100% solvent B [methanol:hexane, 5∶1 (v/v)], with a run time of 30 min and a flow rate of 1.5 ml⋅min^−1^. Pigments were detected by absorbance at 445 nm. The de-epoxidation state of the xanthophyll cycle was calculated as (Z+A)/(V+Z+A).

### Transmission Electron Microscopy of Chloroplasts

The second fully expanded leaves from the top of the plants were randomly selected for microscopic examination. The leaf samples were cut into pieces of approximately 1 mm^2^ and fixed overnight with 4% glutaraldehyde in 0.1 M phosphate-buffered saline (PBS, pH 7.4). The fixed samples were washed three times with the same solution for 10 min each. The samples were then post-fixed in 1% osmium tetroxide in cacodylate buffer for 2 h, and then washed three times in 0.1 M PBS (pH 7.4). The samples were dehydrated in a graded ethanol series (50%, 70%, 90%, and 100%), and then in absolute acetone for 15 min. The samples were embedded in Spurr’s resin, and ultra-thin sections were cut and stained with uranium acetate and lead citrate, in series. The ultra-thin sections were mounted on copper grids and examined with a HITACHI HT7700 transmission electron microscope.

### Statistical Analysis

All treatments and measurements were conducted at least in triplicate. All data were statistically analyzed with statistical software SAS (version 8.0, SAS Institute, Cary, NC) using Duncan’s multiple range test at the *P* less than 0.05 level of significance.

## Results

### Plant Growth

The fresh and dry weights of tomato seedlings were significantly reduced by salinity-alkalinity stress compared with controls after 4 days of treatment (Tables 1 and 2). Reductions in dry leaf and shoot weights were alleviated by addition of exogenous Spd (Table 2). The S treatment had higher R/S than any other treatment (Table 2). There were no significant differences between S and SS treatments with respect to dry root weight and dry stem weight (Table 2). These results indicated that tomato seedling leaf growth was inhibited by salinity-alkalinity stress, and this inhibition was alleviated by exogenous Spd. However, exogenous Spd did not significantly affect the fresh weight measurements of tomato seedlings with or without salinity-alkalinity stress (Table 1).

**Table 1:** Effects of exogenous spermidine on tomato seedling fresh weight under salinity-alkalinity stress.

Treatment	Shoot fresh weight (g)	Root fresh weight (g)	Stem fresh weight (g)	Leaf freshweight (g)	R/S fresh weight
CK	40.726±2.066^a^	6.743±0.276^a^	17.968±1.263^a^	22.759±0.946^a^	0.166±0.007^a^
CS	43.578±3.130^a^	7.400±0.650^a^	18.348±1.737^a^	25.231±1.543^ab^	0.170±0.007^a^
S	22.482±0.711^b^	3.718±0.338^b^	5.649±0.555^b^	16.834±1.232^c^	0.167±0.020^a^
SS	26.050±0.938^b^	3.911±0.272^b^	6.877±0.940^b^	19.172±0.906^bc^	0.150±0.008^a^

**Note:** Data were measured after salinity-alkalinity treatment for 4 days. Each value represents mean ± standard error of four independent experiments (*n* = 4). Different letters indicate significant differences between treatments (*P*<0.05).

**Abbreviations:** CK, 0 mM salinity-alkalinity plus 0 mM Spd; CS, 0 mM salinity-alkalinity plus 0.25 mM Spd; S, 75 mM salinity-alkalinity plus 0 mM Spd; SS, 75 mM salinity-alkalinity plus 0.25 mM Spd.

**Table 2:** Effects of exogenous spermidine on tomato seedling dry weight under salinity-alkalinity stress.

Treatment	Shoot dryweight (g)	Root dryweight (g)	Stem dryweight (g)	Leaf dryweight (g)	R/S dry weight
CK	3.049±0.312^a^	0.396±0.034^a^	0.978±0.139^a^	2.071±0.176^a^	0.131±0.009^b^
CS	3.018±0.250^a^	0.396±0.029^a^	0.979±0.086^a^	2.040±0.164^a^	0.132±0.004^b^
S	1.547±0.116^c^	0.289±0.037^b^	0.451±0.045^b^	1.097±0.084^c^	0.185±0.011^a^
SS	2.216±0.115^b^	0.264±0.023^b^	0.626±0.076^b^	1.591±0.046^b^	0.118±0.006^b^

**Note:** Data were measured after salinity-alkalinity treatment for 4 days. Each value represents mean ± standard error of four independent experiments (*n* = 4). Different letters indicate significant differences between treatments (*P*<0.05).

**Abbreviations:** CK, 0 mM salinity-alkalinity plus 0 mM Spd; CS, 0 mM salinity-alkalinity plus 0.25 mM Spd; S, 75 mM salinity-alkalinity plus 0 mM Spd; SS, 75 mM salinity-alkalinity plus 0.25 mM Spd.

### Gas-Exchange Parameters

**Figure 1:**
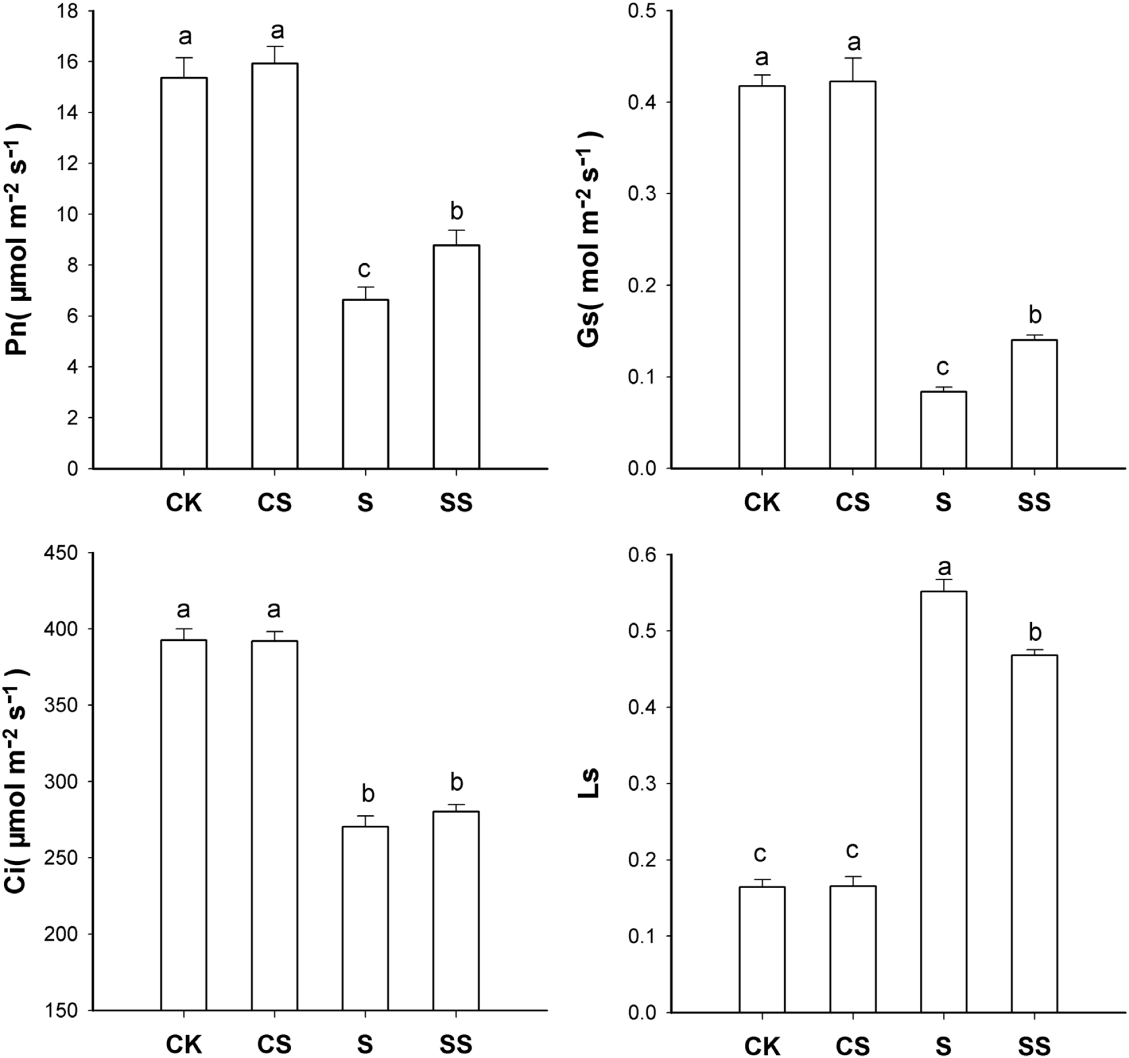
Effects of exogenous spermidine on gas-exchange parameters in tomato seedlings under salinity-alkalinity stress. Data were measured in the second expanded leaves (numbered basipetally) after salinity-alkalinity treatment for 4 days. Each histogram represents the mean ± standard error of four independent experiments (*n* = 4). Different letters indicate significant differences between treatments (*P*<0.05).

Salinity-alkalinity stress significantly reduced the net photosynthetic rate (P_n_), stomatal conductance (G_s_), and C_i_, and increased L_s_, compared with those of controls (Fig. 1). Exogenous Spd alleviated reductions in P_n_ and G_s_, and increase in L_s_. Exogenous Spd had no significant effect on C_i_ under salinity-alkalinity stress (SS treatment) compared with that of S treatment. No significant differences in P_n_, G_s_, C_i_, or L_s_ were detected in CS and CK treatments.

### Chlorophyll Fluorescence

**Figure 2:**
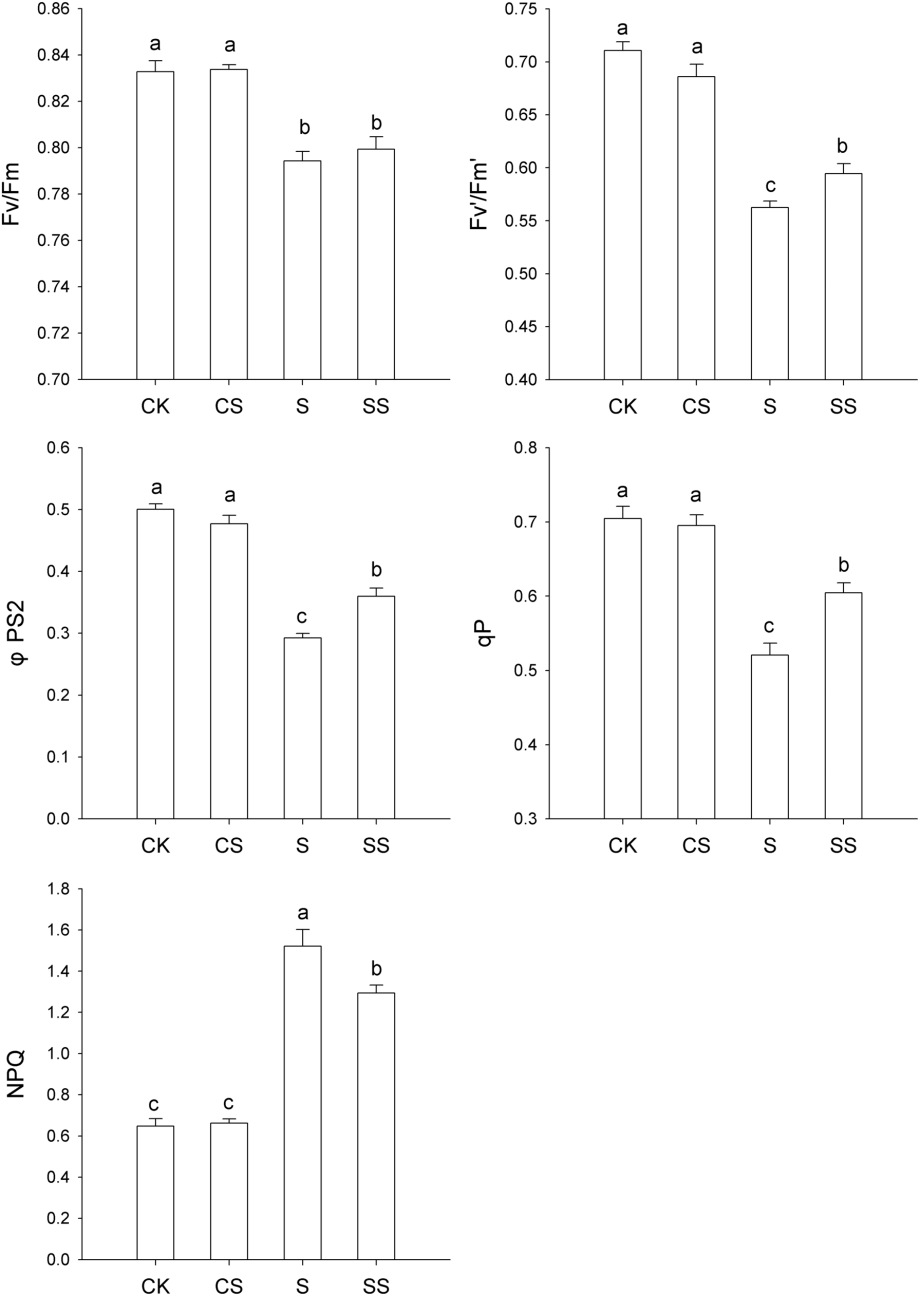
Effects of exogenous spermidine on chlorophyll fluorescence parameters in tomato seedlings under salinity-alkalinity stress. Data were measured in the second expanded leaves (numbered basipetally) after salinity-alkalinity treatment for 4 days. Each histogram represents the mean ± standard error of four independent experiments (*n* = 4). Different letters indicate significant differences between treatments (*P*<0.05).

F_v_′/F_m_′, ΦPSII, and qP were substantially reduced under salinity-alkalinity conditions compared with controls (Fig. 2). The addition of exogenous Spd alleviated these effects (Fig. 2). The maximum quantum yield of PSII (F_v_/F_m_) was reduced in plants subjected to salinity-alkalinity stress. The addition of exogenous Spd did not protect the quantum yield of PSII. Plants subjected to salinity-alkalinity stress had significantly elevated NPQ, and this increase was alleviated by exogenous Spd. Exogenous Spd did not affect F_v_/F_m_, F_v_′/F_m_′, ΦPSII, qP, or NPQ in the absence of salinity-alkalinity stress.

### Xanthophyll Cycle Components

The Z content and the total V+A+Z pool size increased in response to salinity-alkalinity stress, and exogenous Spd induced an even higher increase in the contents of these metabolites. The V content markedly decreased in response to salinity-alkalinity stress, and this decrease was greater in leaves without exogenous Spd than in those with Spd. The (A+Z)/(V+Z+A) ratio increased in response to salinity-alkalinity stress in plants treated with exogenous Spd, but it was lower than in those without Spd. These results indicate that Spd increased the total V+A+Z pool size and the constitutive accumulation of Z in response to salinity-alkalinity stress.

### Ultrastructural Changes in Chloroplasts

**Figure 3:**
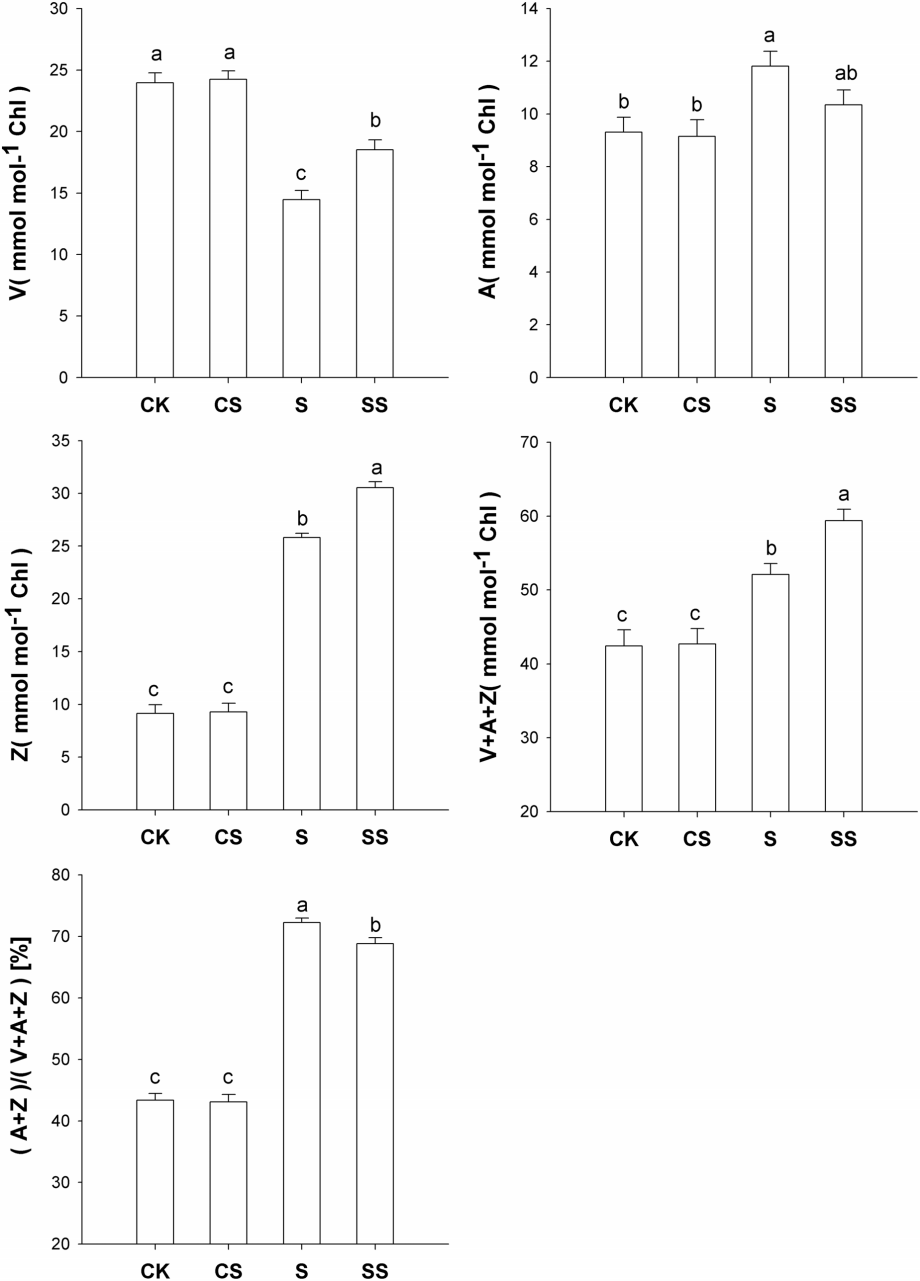
Effects of exogenous spermidine on xanthophyll cycle components in tomato seedlings under salinity-alkalinity stress. Data were measured in the second expanded leaves (numbered basipetally) after salinity-alkalinity treatment for 4 days. Each histogram represents the mean ± standard error of four independent experiments (*n* = 3). Different letters indicate significant differences between treatments (*P*<0.05).

A typical elongated chloroplast was observed in controls (Fig. 3B), which had intact double membranes and a regular arrangement of granal and stromal thylakoids. Under salinity-alkalinity stress (Fig. 3G–I), chloroplasts were swollen and contained more plastoglobuli than those in controls (Fig. 3A–C). The chloroplast membrane structures became dilatant, undefined, and in some cases appeared disintegrated (Fig. 3H). The disoriented lamellar system of the granal and stromal thylakoids became swollen and unclear (Fig. 3I). The numbers of grana were considerably reduced. Exogenous Spd alleviated the salinity-alkalinity-mediated damage of the chloroplast photosynthetic apparatus, and more normal chloroplast ultrastructure was observed (Fig. 3J–L). No significant differences in chloroplast ultrastructure were observed in controls with or without the application of Spd (Fig. 3A–F).

## Discussion

PAs are low molecular weight, ubiquitous, nitrogenous compounds that function in many cellular processes in numerous organisms [28]. Due to their polycationic nature and positive charge at physiological pH, PAs bind to macromolecules such as proteins and nucleic acids [14], [29]. In this way, PAs are involved in regulating the physical and chemical properties of membranes, stabilizing nucleic acid structure and function, and modulating enzyme activities [30]. Chloroplasts are the primary sites of photosynthetic reactions in higher plants. Some types of stress reduce photochemical efficiency and electron transport activity, which might reflect changes in the structure of the photosynthetic apparatus [31]. Previous studies report that PAs stabilize the photosynthetic apparatus in response to stress [15].

In the present study, the deleterious effects of salinity-alkalinity stress on leaf and shoot dry weights in tomato seedlings were partly counteracted by the addition of exogenous Spd (Table 1). The improved dry matter accumulation in shoots and leaves might be associated with Spd-mediated improvement in P_n_ in tomato seedlings under salinity-alkalinity conditions (Fig. 1). A similar result was reported by Zhang et al. in salt-stressed cucumber plants sprayed with Put, which enhanced leaf area, plant height, and fresh and dry weight accumulation [24]. These authors demonstrated that Put likely enhanced photosynthetic production of salt-stressed plants, and this increased production appeared to effectively support the enhance growth. In tomato plants subjected to salinity-alkalinity stress and treated with exogenous Spd, our R/S results showed that most photosynthetic products were utilized to produce shoots rather than roots (Table 2).

Salinity-alkalinity stress reduced P_n_ and G_s_, and increased L_s_ (Fig. 1). Previous studies evaluated the relationship between L_s_ and nonstomatal factors. If both C_i_ and G_s_ decreased, P_n_ was limited primarily by stomatal conductance. If G_s_ decreased but C_i_ did not change or increased, the P_n_ decrease could be ascribed to nonstomatal factors [19], [24], [32], [33]. In the present study, salinity-alkalinity-induced reductions in P_n_ and G_s_ and increase in L_s_ were alleviated by exogenous Spd. However, exogenous Spd had no significant effect on C_i_ with or without stress (Fig. 1). These observed changes in G_s_, C_i_, and L_s_ imply that exogenous Spd alleviates both L_s_ and nonstomatal limitations under salinity-alkalinity conditions.

In general, F_v_/F_m_, F_v_′/F_m_′, ΦPSII, and qP are parameters that reflect photochemical quenching, whereas NPQ reflects nonphotochemical quenching [26]. In this study, the values of F_v_/F_m_, F_v_′/F_m_′, ΦPSII, and qP were reduced by salinity-alkalinity treatment, and these decreases were alleviated by exogenous Spd. A previous report suggested that salt-stress-induced reductions in F_v_′/F_m_′ and ΦPSII might reflect damage to PSII electron transport [19]. These authors demonstrated that inhibition of electron transport may occur during transfer from the primary acceptor plastoquinone (QA) to the secondary acceptor plastoquinone (QB) at the acceptor side of PSII. Although exogenous Spd had no significant effect on F_v_/F_m_ of tomato seedlings with or without salinity-alkalinity stress, it alleviated the stress-induced reductions in F_v_′/F_m_′ and ΦPSII. This result indicates that exogenous Spd alleviates the stress-induced inhibition of photosynthetic electron transport.

**Figure 4:**
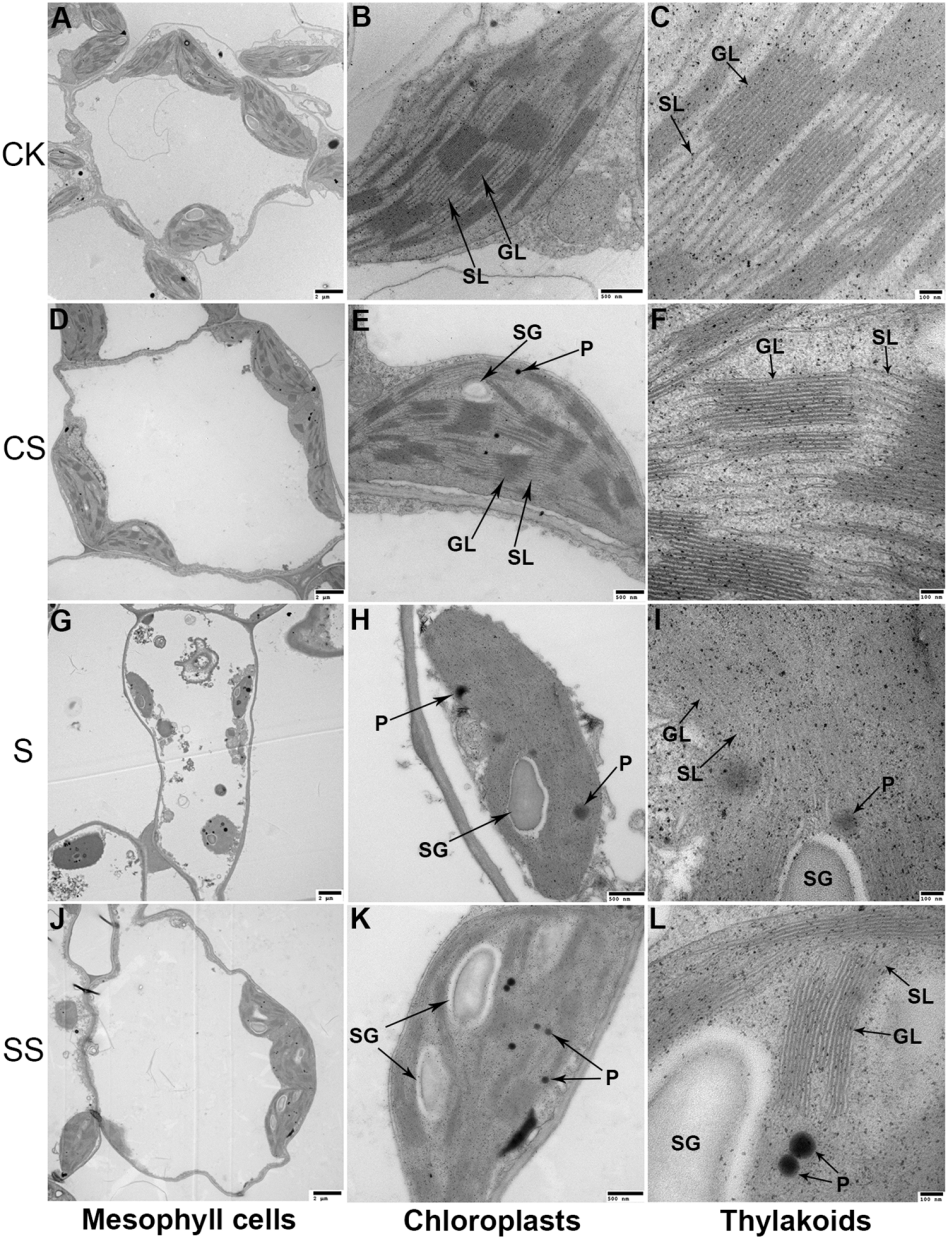
Effects of exogenous spermidine on chloroplast ultrastructure in tomato seedlings under salinity-alkalinity stress. Data were measured in the second expanded leaves (numbered basipetally) after salinity-alkalinity treatment for 4 days. SL, stroma lamellae; GL, grana lamellae; SG, starch grains; P, plastoglobuli. Scale bars for mesophyll cells, chloroplasts, and thylakoids are 2, 0.5, and 0.1 µm, respectively.

Exogenous Spd alleviated the salinity-alkalinity-induced decrease in qP and increase in NPQ. This suggests that exogenous Spd may increase qP at the expense of NPQ under stress conditions, thereby mitigating the dissipation of excitation energy in the PSII antennae [34]. This is supported by the observation that exogenous Spd improved P_n_ and decreased the de-epoxidation status of the xanthophyll cycle in tomato seedlings under salinity-alkalinity stress (Fig. 2). The xanthophyll cycle serves as an essential photoprotective process in the dissipation of excess energy [35], [36]. This thermal dissipation is an important function of NPQ to alleviate excess excitation energy on PSII reaction centers by converting excess excitation energy into heat [37]. Previous work showed that, to a certain extent, severe stress might result in higher levels of NPQ and (A+Z)/(V+Z+A) [38], [39]. Consequently, under salinity-alkalinity conditions, leaves treated with Spd suffered less photoinhibition than those without Spd. Exogenous Spd may protect the photosynthetic apparatus against over-excitation, perhaps by preventing a loss of thylakoid membrane integrity [40]. The observed chloroplast ultrastructures support the proposal that exogenous Spd alleviates injury to the photosynthetic membrane caused by salinity-alkalinity stress (Fig. 4J–L). This result may be attributed to increases in the total V+A+Z pool size and the Spd-induced increases in Z content.

Z serves another important function as an antioxidant in the lipid phase of the thylakoid membrane to block photo-oxidative stress [21]. Photoinhibition occurs even under moderate light conditions if excess light energy cannot be appropriately dissipated [41]. ^1^O_2_ is one of the most important ROS involved in photoinhibition and photo-oxidative damage [42]. Nonprotein-bound Z enhances photoprotection by blocking the formation of ^1^O_2_ and ^1^O_2_-related oxidation products [43], [44], which reduces lipid peroxidation. ROS scavenging protects chloroplasts from direct injury and diminishes photon (electron)-related stress [7]. In our study, exogenous Spd increased the amount of Z under salinity-alkalinity stress, which stabilized thylakoid membrane structure and improved photosynthetic function.

Chloroplasts are crucial plant organelles because they are the site of photosynthesis. Chloroplast ultrastructure can be substantially disrupted by salinity stress [45]. In the present study, increased numbers of plastoglobuli were observed in mesophyll cells subjected to salinity-alkalinity stress, and some of these were larger than those in controls (Fig. 4G–I). Increases in plastoglobuli numbers may be caused by thylakoid membrane degradation [46], and individual plastoglobuli may be larger when chloroplasts are under oxidative stress [47]. Chloroplasts are a major source of ROS, including ^1^O_2_, OH•, and H_2_O_2_ [7], which cause membrane lipid peroxidation and cellular abnormalities when plants are exposed to environmental stresses [48]. Tian et al. showed that exogenous Spd enhanced the activities of ascorbate peroxidase (APX), superoxide dismutase (SOD), peroxidase (POD), and catalase when cucumber seedlings were subjected to high temperatures [49]. Exogenous Spm alleviated salt-induced chloroplast membrane injury by increasing the levels of antioxidant metabolites (including dehydro-L-ascorbic acid, ascorbic acid, glutathione-S-S-glutathione, and glutathione-stimulating hormone) and enzyme activities (including SOD, POD, and APX) in chloroplasts [16]. PAs also bind membrane proteins, including polypeptides of the light-harvesting complex (LHC) [50]. Exogenous PAs with a high net positive charge can stabilize PSII proteins such as D1 and D2 under photoinhibition conditions [51]. PA binding to membrane proteins may provide stability for protein structure during stress, and consequently preserve photosynthetic activity. Our results indicate that Spd may serve a protective role in chloroplasts by enhancing ROS scavenging and stabilizing photosynthetic membrane proteins.

In conclusion, our results show that salinity-alkalinity stress induces photoinhibition and photodamage in tomato seedlings. Exogenous Spd alleviates the stress-induced inhibition of plant shoot growth and P_n_ by reducing ROS and stabilizing thylakoid membrane structure. Exogenous Spd indirectly improves total V+A+Z pool size, especially the amount of Z, which acts as an antioxidant in the lipid phase of the thylakoid membrane to block photo-oxidative stress. However, under salinity-alkalinity stress, Spd-induced increases in Z content did not increase NPQ compared with that in seedlings without Spd. This may be because Spd protects leaves from light-induced damage due to qP, which differs from heat dissipation.

## References

[1] Zhang Y, Hu XH, Shi Y, Zou ZR, Yan F, et al (2013) Beneficial role of exogenous spermidine on nitrogen metabolism in tomato seedlings exposed to saline-alkaline stress. Journal of the American Society for Horticultural Science 138: 38–49.

[2] Hu X, Zhang Y, Shi Y, Zhang Z, Zou Z, et al (2012) Effect of exogenous spermidine on polyamine content and metabolism in tomato exposed to salinity-alkalinity mixed stress. Plant physiology and biochemistry: PPB/Societe francaise de physiologie vegetale 57: 200–209. 10.1016/j.plaphy.2012.05.015 22738864

[3] Yang J, Zheng W, Tian Y, Wu Y, Zhou D (2011) Effects of various mixed salt-alkaline stresses on growth, photosynthesis, and photosynthetic pigment concentrations of Medicago ruthenica seedlings. Photosynthetica 49: 275–284.

[4] Li R, Shi F, Fukuda K, Yang Y (2010) Effects of salt and alkali stresses on germination, growth, photosynthesis and ion accumulation in alfalfa (Medicago sativa L.). Soil Science & Plant Nutrition 56: 725–733.

[5] Deng C, Zhang G, Pan X (2011) Photosynthetic Responses in Reed (Phragmites australis (CAV.) TRIN. ex Steud.) Seedlings Induced by Different Salinity-Alkalinity and Nitrogen levels. Journal of Agricultural Science and Technology 13: 687–699.

[6] Yadav DK, Pospíšil P (2012) Evidence on the formation of singlet oxygen in the donor side photoinhibition of photosystem II: EPR spin-trapping study. PloS one 7: e45883. 23049883 10.1371/journal.pone.0045883 PMC3458798

[7] Asada K (2006) Production and scavenging of reactive oxygen species in chloroplasts and their functions. Plant physiology 141: 391–396. 16760493 10.1104/pp.106.082040 PMC1475469

[8] Murata N, Takahashi S, Nishiyama Y, Allakhverdiev SI (2007) Photoinhibition of photosystem II under environmental stress. Biochimica et Biophysica Acta (BBA)-Bioenergetics 1767: 414–421. 17207454 10.1016/j.bbabio.2006.11.019

[9] Mittal S, Kumari N, Sharma V (2012) Differential response of salt stress on Brassica juncea: Photosynthetic performance, pigment, proline, D1 and antioxidant enzymes. Plant Physiology and Biochemistry 54: 17–26. 22369937 10.1016/j.plaphy.2012.02.003

[10] Bouchereau A, Aziz A, Larher F, Martintanguy J (1999) Polyamines and environmental challenges: recent development. Plant Science 140: 103–125.

[11] Liu Y, Gu D, Wu W, Wen X, Liao Y (2013) The Relationship between Polyamines and Hormones in the Regulation of Wheat Grain Filling. PloS one 8: e78196. 24205154 10.1371/journal.pone.0078196 PMC3812141

[12] Ha HC, Sirisoma NS, Kuppusamy P, Zweier JL, Woster PM, et al (1998) The natural polyamine spermine functions directly as a free radical scavenger. Proceedings of the National Academy of Sciences 95: 11140–11145. 10.1073/pnas.95.19.11140 PMC21609 9736703

[13] Bellé NAV, Dalmolin GD, Fonini G, Rubin MA, Rocha JBT (2004) Polyamines reduces lipid peroxidation induced by different pro-oxidant agents. Brain research 1008: 245–251. 15145762 10.1016/j.brainres.2004.02.036

[14] Groppa MD, Benavides MP (2008) Polyamines and abiotic stress: recent advances. Amino acids 34: 35–45. 17356805 10.1007/s00726-007-0501-8

[15] Demetriou G, Neonaki C, Navakoudis E, Kotzabasis K (2007) Salt stress impact on the molecular structure and function of the photosynthetic apparatus–the protective role of polyamines. Biochimica et Biophysica Acta (BBA)-Bioenergetics 1767: 272–280. 17408588 10.1016/j.bbabio.2007.02.020

[16] Shu S, Yuan LY, Guo SR, Sun J, Yuan YH (2013) Effects of exogenous spermine on chlorophyll fluorescence, antioxidant system and ultrastructure of chloroplasts in Cucumis sativus L. under salt stress. Plant physiology and biochemistry: PPB/Societe francaise de physiologie vegetale 63: 209–216. 10.1016/j.plaphy.2012.11.028 23291654

[17] Shu S, Guo SR, Sun J, Yuan LY (2012) Effects of salt stress on the structure and function of the photosynthetic apparatus in Cucumis sativus and its protection by exogenous putrescine. Physiologia plantarum 146: 285–296. 22452600 10.1111/j.1399-3054.2012.01623.x

[18] Li Y, Shi G, Wang H, Zhao J, Yuan Q (2009) Exogenous spermidine can mitigate the poison of cadmium in nymphoides peltatum. Chinese Bulletin of Botany 44: 571–577.

[19] Sheng S (2012) Effects of exogenous spermidine on photosynthesis, xanthophyll cycle and endogenous polyamines in cucumber seedlings exposed to salinity. African Journal of Biotechnology 11: 6064–6074.

[20] Demmigadams B, Adams III WW (2006) Photoprotection in an ecological context: the remarkable complexity of thermal energy dissipation. The New phytologist 172: 11–21. 16945085 10.1111/j.1469-8137.2006.01835.x

[21] Jahns P, Holzwarth AR (2012) The role of the xanthophyll cycle and of lutein in photoprotection of photosystem II. Biochimica et biophysica acta 1817: 182–193. 21565154 10.1016/j.bbabio.2011.04.012

[22] Demmigadams B, Adams III WW (1996) The role of xanthophyll cycle carotenoids in the protection of photosynthesis. Trends in Plant science 1: 21–26.

[23] Zhang R, Kramer DM, Cruz JA, Struck KR, Sharkey TD (2011) The effects of moderately high temperature on zeaxanthin accumulation and decay. Photosynthesis research 108: 171–181. 21785990 10.1007/s11120-011-9672-y

[24] Zhang RH, Li J, Guo SR, Tezuka T (2009) Effects of exogenous putrescine on gas-exchange characteristics and chlorophyll fluorescence of NaCl-stressed cucumber seedlings. Photosynthesis research 100: 155–162. 19507048 10.1007/s11120-009-9441-3

[25] Farquhar GD, Sharkey TD (1982) Stomatal conductance and photosynthesis. Annual Review of Plant Physiology 33: 317–345.

[26] Baker NR (2008) Chlorophyll fluorescence: a probe of photosynthesis in vivo. Annual Review of Plant Biology 59: 89–113. 10.1146/annurev.arplant.59.032607.092759 18444897

[27] Chen C, Zhang D, Li P, Ma F (2012) Partitioning of absorbed light energy differed between the sun-exposed side and the shaded side of apple fruits under high light conditions. Plant physiology and biochemistry: PPB/Societe francaise de physiologie vegetale 60: 12–17. 10.1016/j.plaphy.2012.07.016 22892330

[28] Hussain SS, Ali M, Ahmad M, Siddique KH (2011) Polyamines: natural and engineered abiotic and biotic stress tolerance in plants. Biotechnology advances 29: 300–311. 21241790 10.1016/j.biotechadv.2011.01.003

[29] Childs AC, Mehta DJ, Gerner EW (2003) Polyamine-dependent gene expression. Cellular and molecular life sciences: CMLS 60: 1394–1406. 12943227 10.1007/s00018-003-2332-4 PMC11138590

[30] Galston AW, Sawhney RK (1990) Polyamines in plant physiology. Plant physiology 94: 406–410. 11537482 10.1104/pp.94.2.406 PMC1077246

[31] Parida A, Das A, Mittra B (2003) Effects of NaCl stress on the structure, pigment complex composition, and photosynthetic activity of mangrove Bruguiera parviflora chloroplasts. Photosynthetica 41: 191–200.

[32] Liu ZX, Bie ZL, Huang Y, Zhen A, Lei B, et al (2012) Grafting onto Cucurbita moschata rootstock alleviates salt stress in cucumber plants by delaying photoinhibition. Photosynthetica 50: 152–160.

[33] Begcy K, Mariano ED, Gentile A, Lembke CG, Zingaretti SM, et al (2012) A novel stress-induced sugarcane gene confers tolerance to drought, salt and oxidative stress in transgenic tobacco plants. PloS one 7: e44697. 22984543 10.1371/journal.pone.0044697 PMC3439409

[34] Horton P, Ruban A, Walters R (1996) Regulation of light harvesting in green plants. Annual review of plant biology 47: 655–684. 10.1146/annurev.arplant.47.1.655 15012304

[35] Bilger W, Björkman O (1990) Role of the xanthophyll cycle in photoprotection elucidated by measurements of light-induced absorbance changes, fluorescence and photosynthesis in leaves of Hedera canariensis. Photosynthesis research 25: 173–185. 24420348 10.1007/BF00033159

[36] Goss R, Jakob T (2010) Regulation and function of xanthophyll cycle-dependent photoprotection in algae. Photosynthesis research 106: 103–122. 20224940 10.1007/s11120-010-9536-x

[37] Nilkens M, Kress E, Lambrev P, Miloslavina Y, Muller M, et al (2010) Identification of a slowly inducible zeaxanthin-dependent component of non-photochemical quenching of chlorophyll fluorescence generated under steady-state conditions in Arabidopsis. Biochimica et biophysica acta 1797: 466–475. 20067757 10.1016/j.bbabio.2010.01.001

[38] Yin Y, Li S, Liao W, Lu Q, Wen X, et al (2010) Photosystem II photochemistry, photoinhibition, and the xanthophyll cycle in heat-stressed rice leaves. Journal of plant physiology 167: 959–966. 20417985 10.1016/j.jplph.2009.12.021

[39] Guo YP, Guo DP, Zhou HF, Hu MJ, Shen YG (2006) Photoinhibition and xanthophyll cycle activity in bayberry (Myrica rubra) leaves induced by high irradiance. Photosynthetica 44: 439–446.

[40] Ogweno JO, Hu WH, Song XS, Shi K, Mao WH, et al (2010) Photoinhibition-induced reduction in photosynthesis is alleviated by abscisic acid, cytokinin and brassinosteroid in detached tomato leaves. Plant Growth Regulation 60: 175–182.

[41] Demmigadams B, Adams III WW (1992) Photoprotection and other responses of plants to high light stress. Annual review of plant biology 43: 599–626.

[42] Triantaphylidès C, Krischke M, Hoeberichts FA, Ksas B, Gresser G, et al (2008) Singlet oxygen is the major reactive oxygen species involved in photooxidative damage to plants. Plant physiology 148: 960–968. 18676660 10.1104/pp.108.125690 PMC2556806

[43] Wrona M, Różanowska M, Sarna T (2004) Zeaxanthin in combination with ascorbic acid or α-tocopherol protects ARPE-19 cells against photosensitized peroxidation of lipids. Free Radical Biology and Medicine 36: 1094–1101. 15082063 10.1016/j.freeradbiomed.2004.02.005

[44] Havaux M, DallOsto L, Bassi R (2007) Zeaxanthin has enhanced antioxidant capacity with respect to all other xanthophylls in Arabidopsis leaves and functions independent of binding to PSII antennae. Plant physiology 145: 1506–1520. 17932304 10.1104/pp.107.108480 PMC2151694

[45] Paramonova N, Shevyakova N, Kuznetsov VV (2004) Ultrastructure of chloroplasts and their storage inclusions in the primary leaves of Mesembryanthemum crystallinum affected by putrescine and NaCl. Russian Journal of Plant Physiology 51: 86–96.

[46] Naeem MS, Warusawitharana H, Liu H, Liu D, Ahmad R, et al (2012) 5-aminolevulinic acid alleviates the salinity-induced changes in Brassica napus as revealed by the ultrastructural study of chloroplast. Plant physiology and biochemistry: PPB/Societe francaise de physiologie vegetale 57: 84–92. 10.1016/j.plaphy.2012.05.018 22695221

[47] Austin JR, Frost E, Vidi PA, Kessler F, Staehelin LA (2006) Plastoglobules are lipoprotein subcompartments of the chloroplast that are permanently coupled to thylakoid membranes and contain biosynthetic enzymes. The Plant Cell Online 18: 1693–1703. 10.1105/tpc.105.039859 PMC1488921 16731586

[48] Gill SS, Tuteja N (2010) Reactive oxygen species and antioxidant machinery in abiotic stress tolerance in crop plants. Plant Physiology and Biochemistry 48: 909–930. 20870416 10.1016/j.plaphy.2010.08.016

[49] Tian J, Wang LP, Yang YJ, Sun J, Guo SR (2012) Exogenous spermidine alleviates the oxidative damage in cucumber seedlings subjected to high temperatures. Journal of the American Society for Horticultural Science 137: 11–19.

[50] Hamdani S, Yaakoubi H, Carpentier R (2011) Polyamines interaction with thylakoid proteins during stress. Journal of Photochemistry and Photobiology B: Biology 104: 314–319. 10.1016/j.jphotobiol.2011.02.007 21377374

[51] Hamdani S, Gauthier A, Msilini N, Carpentier R (2011) Positive charges of polyamines protect PSII in isolated thylakoid membranes during photoinhibitory conditions. Plant and cell physiology 52: 866–873. 21471122 10.1093/pcp/pcr040

